# Supplementary material for: The Systems Biology Research Tool: evolvable open-source software
Source: BMC Syst Biol. 2008 Jun 29;2:55. doi: 10.1186/1752-0509-2-55 (PMC2446383; doi:10.1186/1752-0509-2-55)
Supplement: Additional file 1 — SBRT Archive. An archive of the current version of the Systems Biology Research Tool. [file 1752-0509-2-55-S1.zip › sbrt-1.4.0/doc/developers_guide/api/sbrt/kernel/fba/ChemRxn.html]

ChemRxn


|  |  |  |
| --- | --- | --- |
| **Overview** \| **Package** \| **Class** \| **Use** \| **Tree** \| **Deprecated** \| **Index** \| **Help** | |  |
| **PREV CLASS**   **NEXT CLASS** | **FRAMES**    **NO FRAMES**     **All Classes** |  |
| SUMMARY: NESTED \| FIELD \| CONSTR \| METHOD | DETAIL: FIELD \| CONSTR \| METHOD |  |


---


## sbrt.kernel.fba Interface ChemRxn

**All Known Implementing Classes:**: AbstractRxn, ExchangeRxn, IrrevRxn, ReversibleRxn, SinkRxn, SourceRxn

---

``` public interface ChemRxn ```

This interface is used to represent simple chemical reactions. It was intended
primarily for use in Flux Balance Analysis, but may have other applications as well.

A chemical reaction is composed of reactants and products, which can each be
represented as linear combinations of chemical species. Some chemical
reactions are catalyzed, and some are even referred to by a specific name.
This interface captures these basic facts about chemical reactions.

**Author:**
:   This interface was written and documented by Jeremiah Wright
    while in the Wagner Lab.

---

| **Method Summary** | |
| --- | --- |
| `boolean` | `equals(java.lang.Object obj)`             Indicates if this chemical reaction is equal to the provided object. |
| `LinearComb<java.lang.String>` | `getProducts()`             Returns the products of this chemical reaction as a linear combination of chemical species. |
| `LinearComb<java.lang.String>` | `getReactants()`             Returns the reactants of this chemical reaction as a linear combination of chemical species. |
| `int` | `hashCode()`             Returns a content-based hash code for this chemical reaction. |
| `boolean` | `isEquivalent(ChemRxn rxn)`             Indicates if this chemical reaction is stoichiometrically equivalent to the provided reaction. |
| `boolean` | `isReverse(ChemRxn rxn)`             Indicates if the provided chemical reaction is the reverse of this reaction. |
| `java.lang.String` | `toString()`             Returns an appropriate string representation of this chemical reaction. |

**Method Detail**

### getReactants

```
LinearComb<java.lang.String> getReactants()
```

:   Returns the reactants of this chemical reaction as a
    linear combination of chemical species.

    :   **Returns:**: the reactants of this chemical reaction as a linear combination of chemical species.

---


### getProducts

```
LinearComb<java.lang.String> getProducts()
```

:   Returns the products of this chemical reaction as a
    linear combination of chemical species.

    :   **Returns:**: the products of this chemical reaction as a linear combination of chemical species.

---


### isEquivalent

```
boolean isEquivalent(ChemRxn rxn)
```

:   Indicates if this chemical reaction is stoichiometrically
    equivalent to the provided reaction.

For the provided reaction to be stoichiometrically equivalent to this
reaction,
`getReactants().equals(rxn.getReactants())` and
`getProducts().equals(rxn.getProducts())` must both return
`true`.

Note: The way equivalence is defined here is very restrictive.
By this definition, the reactions (2) H_2_ + O_2_
→ (2) H_2_O and H_2_ +
(½) O_2_ → H_2_O are not equivalent, because
their stoichiometric coefficients are different.

:   **Parameters:**: `rxn` - the reaction to test for equivalence. **Returns:**: `true` if both reactions are equivalent; `false` otherwise.


---


### isReverse

```
boolean isReverse(ChemRxn rxn)
```

:   Indicates if the provided chemical reaction is the reverse
    of this reaction.

    For the provided reaction to be the reverse of this reaction,
    `getReactants().equals(rxn.getProducts())` and
    `getProducts().equals(rxn.getReactants())` must
    both return `true`.

    Note: The way reverse reactions are defined here is very restrictive.
    By this definition, the reactions (2) H_2_ + O_2_
    → (2) H_2_O and H_2_O → H_2_ +
    (½) O_2_ are not the reverse of each other, because
    their stoichiometric coefficients are different.

    :   **Parameters:**: `rxn` - the reaction to test. **Returns:**: `true` if the provided reaction is the reverse of this reaction; `false` otherwise.

---


### toString

```
java.lang.String toString()
```

:   Returns an appropriate string representation of this chemical reaction.

    :   **Overrides:**: `toString` in class `java.lang.Object`
    :   **Returns:**: an appropriate string representation of this chemical reaction.

---


### equals

```
boolean equals(java.lang.Object obj)
```

:   Indicates if this chemical reaction is equal to the provided object.

    For a reaction `r` to be equal to this reaction,
    `isEquivalent(r)` must return `true` and both reactions must
    have the same name and catalyst.

    :   **Overrides:**: `equals` in class `java.lang.Object`
    :   **Parameters:**: `obj` - the reference object with which to compare. **Returns:**: `true` if this reaction is equal to the provided provided object; `false` otherwise. **See Also:**: `isEquivalent(ChemRxn)`

---


### hashCode

```
int hashCode()
```

:   Returns a content-based hash code for this chemical reaction.

    :   **Overrides:**: `hashCode` in class `java.lang.Object`
    :   **Returns:**: a content-based hash code for this chemical reaction.


---


|  |  |  |
| --- | --- | --- |
| **Overview** \| **Package** \| **Class** \| **Use** \| **Tree** \| **Deprecated** \| **Index** \| **Help** | |  |
| **PREV CLASS**   **NEXT CLASS** | **FRAMES**    **NO FRAMES**     **All Classes** |  |
| SUMMARY: NESTED \| FIELD \| CONSTR \| METHOD | DETAIL: FIELD \| CONSTR \| METHOD |  |


---
